# American Medical Association

**Published:** 1884-09

**Authors:** Jno. S. Marshall


### AMERICAN MEDICAL ASSOCIATION.

### SECTION ON ORAL AND DENTAL SURGERY.

### SESSION OF MAY 8, 1884.

REPORTED FOR THE INDEPENDENT PRACTITIONER BY JNO. S. MARSHALL, M. D.

(Continued from page 454.)

### SYNOPSIS.

#### SPONGE-GRAFTING, BY EDWARD C. BRIGGS, M. D., BOSTON, MASS.

The author opened his paper by referring to the history of the facts which first suggested the idea of sponge-grafting, viz: the experiments of Mr. D. J. Hamilton, lecturer on pathology in the Edinburgh School of Medicine, 1879, "On the Process of Healing," and published in the *Journal of Anatomy and Physiology*, vol. xiii. The statement was there made, after having been proved experimentally, that in a granulating surface there were new vessels formed, but that the superficial capillaries of the part were pushed upwards as granulating loops, by the action of the heart, the projection being permitted from the fact that the restraining influence of the epethelial covering had been removed. Two years later (November number *Edinburgh Medical Journal*), he published an article on "Sponge-grafting," and there states that while gathering information for the former paper, he was led to believe the process of vascularization, as seen in a granulating surface, was similar to that which occurs when a blood-clot or a fibrinous exudate is replaced by a vascular cicatricial tissue, the blood-clot or exudate acting simply as a mechanical support to the projecting capillary loops. With this idea in view he searched for some artificial substance to replace the blood-clot, and finally hit upon sponge as best filling the requirements, by being very porous, and it would thus imitate the interstices of the fibrinous net-work of a blood-clot, and being an animal tissue would, under favorable conditions, be absorbed, like cat-gut or a blood-clot.

The first experiment was with a chronic ulcer of the leg. A piece of sponge, after being prepared as will be described later, was fitted to the ulcerated surface, and its edges tucked under the indurated margins of the ulcer, and covered with a simple antiseptic dressing and bandage. Next day, on dressing, he found the sponge partly filled with purulent discharge. The second day there was a *distinct putrefactive odor;* the sponge was washed with carbolic acid solution, and it then appeared slightly red at the thinnest parts, and the margins of the ulcer reached further in over the sponge. The third day there were points of adhesion between the sponge and granulating surface, and at one point it gave signs of beginning to dissolve. On the fifth day the thinnest parts of the sponge were growing hard, and seemed to be filled with organizing tissue, and on pricking these points they bled freely, proving that the blood-vessels had already penetrated its pores. The sponge originally fitted to the ulcer was five inches in diameter, but at the end of three months only a small piece could be seen.

This experiment proved that in such cases the sponge would act better than the blood-clot, for while the latter would have been destroyed by the putrescent condition of the wound, the sponge, from its greater resistance, did not seem to be the least affected. Since the experiments of Mr. Hamilton, surgeons all over the world have been working in this direction, and many successful cases have from time to time been reported.

These reports suggested the idea that morbid conditions of the mouth, associated with loss of the soft tissues, might likewise be benefited by sponge-grafting. In giving my experiments I will state what I then did, and not what I would now do, with the advantage of personal experience.

Case 1. Mr. N. G. B. had been annoyed for three years by a deep depression over the roots of the right superior first bicuspid, the result of a severe pericementitis, the chief annoyance being the collection of food in the depression, and the retention of the pus. It was not a simple fistula, but a cavity one-half an inch deep, and three-sixteenths of an inch in diameter. He had tried various ways to restore the lost tissue, or narrow the opening. Had created a fresh blood-clot to fill the opening, but this also failed. January 26, 1884, he fitted a piece of sponge to the cavity, previously prepared according to the formula of Dr. Edward Borck, of St. Louis, which is as follows:

"Soak a piece of sponge for three or four days in a twenty per cent. solution of hydrochloric acid. Squeeze it dry and place it in a solution of iodoform and ether (ʒj to ℥j), evaporate the ether, and keep in an air-tight vessel."

Next day, on attempting to remove the sponge, I found to my surprise resistance to my efforts, but persisting, I pulled it out, and discovered a fresh granulating surface, which bled freely. I packed the cavity with fresh sponge. The second day I again removed the sponge, and found the same resistance, and the cavity partially filled with fresh granulations. This treatment was pursued for two months; after the first week I saw the patient only twice a week.

The sponge would apparently become attached for a day or two, and then work out, and this necessitated the application of a new piece. Adhesion never became as firm as with the first piece introduced, and at no time was there any evidence that the sponge would become organized. It acted either as a stimulant or as a support to the granulations, for it accomplished what I had failed to do by any other means. The case now presents only a small fistulous opening three-sixteenths of an inch in depth.

Case 2. About April 1st, encouraged by the success of this first experiment, I undertook a second case in the person of Mr. J. B. Three years previously a separation had been made between the first and second right superior molars, for a dental operation, from which time trouble began in the gum between these teeth. While in Honolulu, a year later, the annoyance was so great that he consulted a dentist, who lanced deeply between the teeth. After the closing of the wound the tissues contracted, making the trouble worse. When I saw the patient, a year ago, there was a deep depression between the teeth, the gum sore, and bleeding freely on irritation. The annoyance was very great, and sometimes caused severe pain. Contouring the teeth was suggested, to protect the gum, but the teeth being very sensitive and the operation likely to be long and tedious, the patient declined.

Treatment was begun April 7th, and at this time the approximal surfaces of the roots were denuded to nearly two-thirds their length. The surface of the gum was wiped with deliquesced crystals of carbolic acid, and the slough removed to give a fresh, healthy surface to act upon.

The sponge for this case was prepared as follows: It was first cleansed of calcareous matter by washing in dilute aqua regia, dried and placed for twenty-four hours in a dilute solution of tincture of iodine, squeezed dry and put in a saturated aqueous solution of boracic acid, when it was ready for use.

To prevent displacement of the sponge and to protect the gum, I made a hard rubber cap to arch over the opening between the teeth, and to rest upon the gum on either side. The sponge being fitted, the inside of the rubber cap was painted with a solution of resin, and put in position. Three days after I removed the cap, syringed the parts, without removing the sponge, with a 1 to 200 solution of carbolic acid, and replaced the cap.

At the next dressing, three days later, I removed the sponge and found it attached only at one side, but the cavity at this point had already begun to fill up a little, and thinking that the sponge as prepared for the first case acted better, I returned to its use. The patient has been seen regularly twice a week, and is progressing very favorably, the cavity filling up as rapidly as could be desired.

The paper closed with several hints relative to the treatment of future cases, viz:

Be sure the parts are thoroughly cleaned. Shape the sponge accurately to the position it is to occupy, not forgetting to allow for the swelling.

Keep pressure upon the sponge for a day or two, to aid in securing its attachment. Disturb the sponge as little as possible, and simply wash away the discharge with some antiseptic fluid.

With regard to its application, he thought it would prove useful not only in such cases as he had described, but in all cases where it was desirable to reproduce soft tissues, and suggested its use in cases of pyorrhœa alveolaris, with loss of tissue about the roots of the teeth.

DISCUSSION.

*Dr. Williams.*—It has been my privilege to see the cases upon which Dr. Briggs operated by sponge-grafting, and I have noted the marked improvement. It is certainly new in its application to the conditions mentioned in the paper, and I think its usefulness in this direction will be very great.

*Dr. Allport.*—I desire to express my thanks to Dr. Briggs for the paper he has read, and I intend to try the sponge-graft on the first case that presents in which there is reasonable hope of success.

*Dr. Briggs.*—I am of the opinion that the sponge-graft may be successfully used in closing certain cases of cleft palate.

